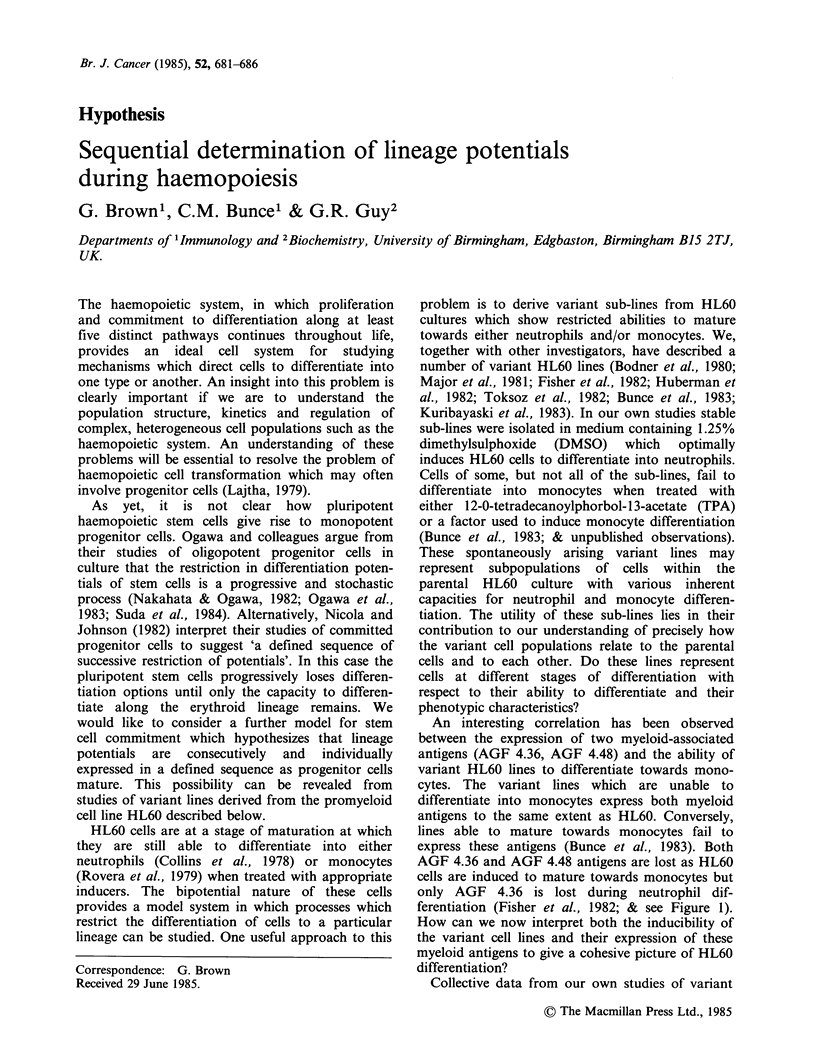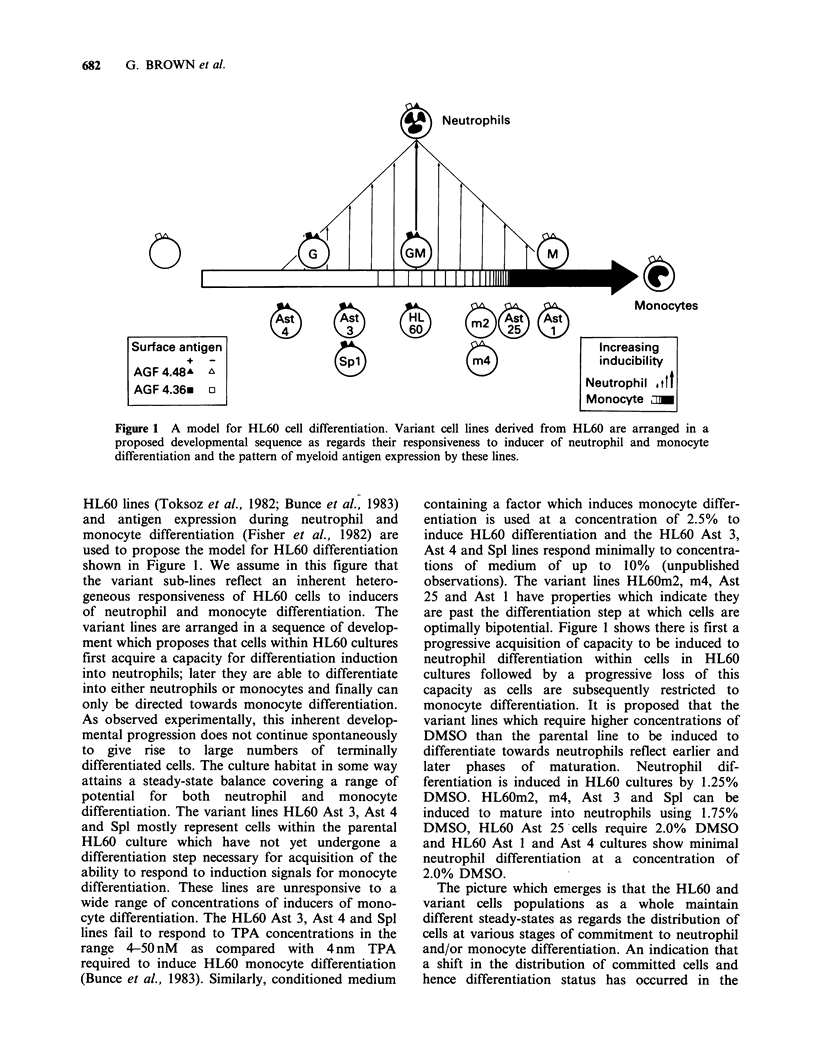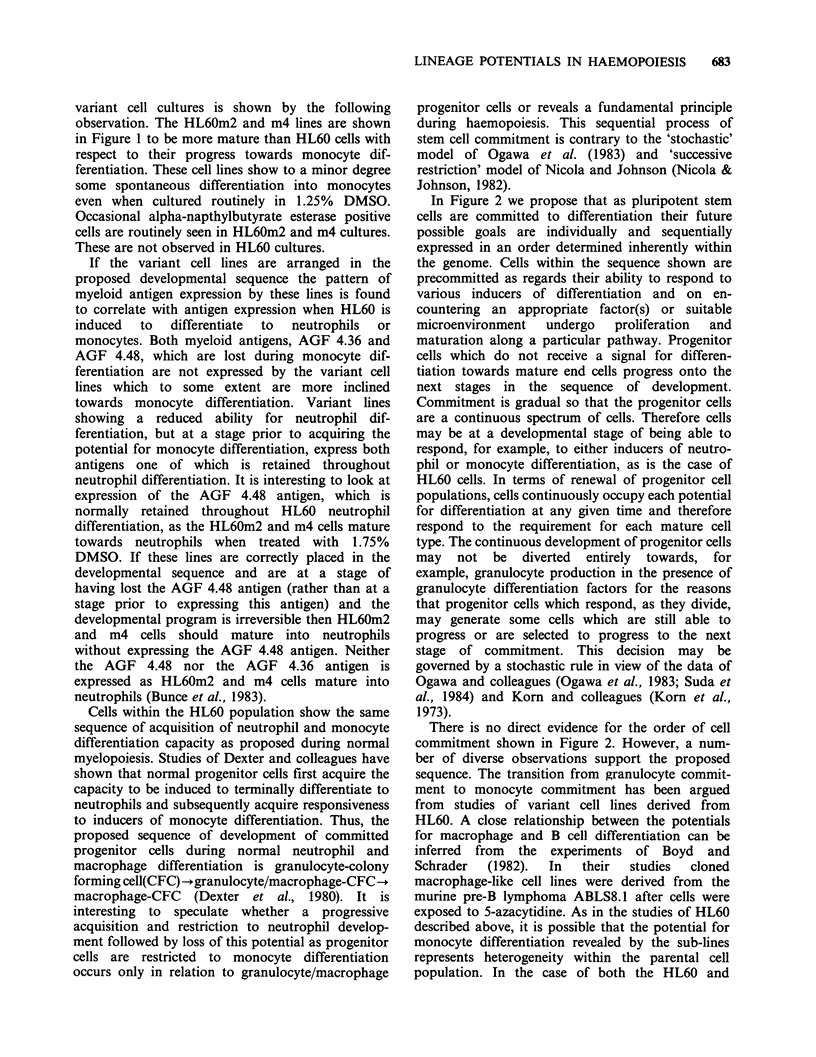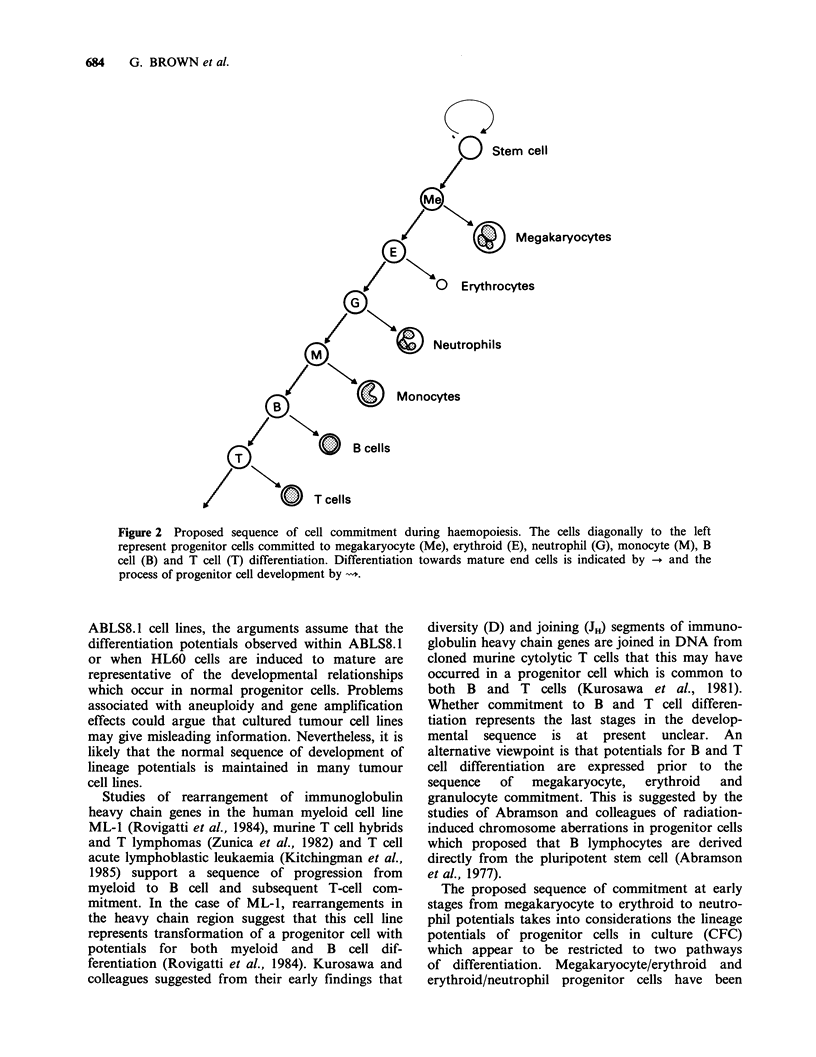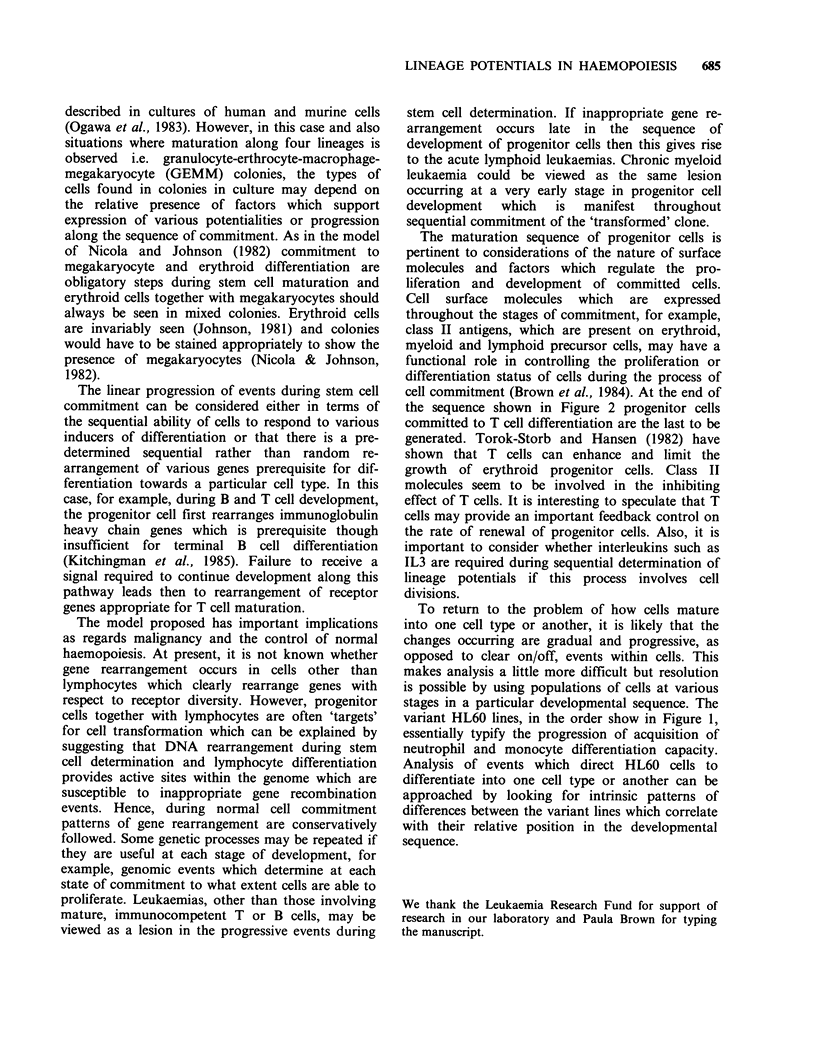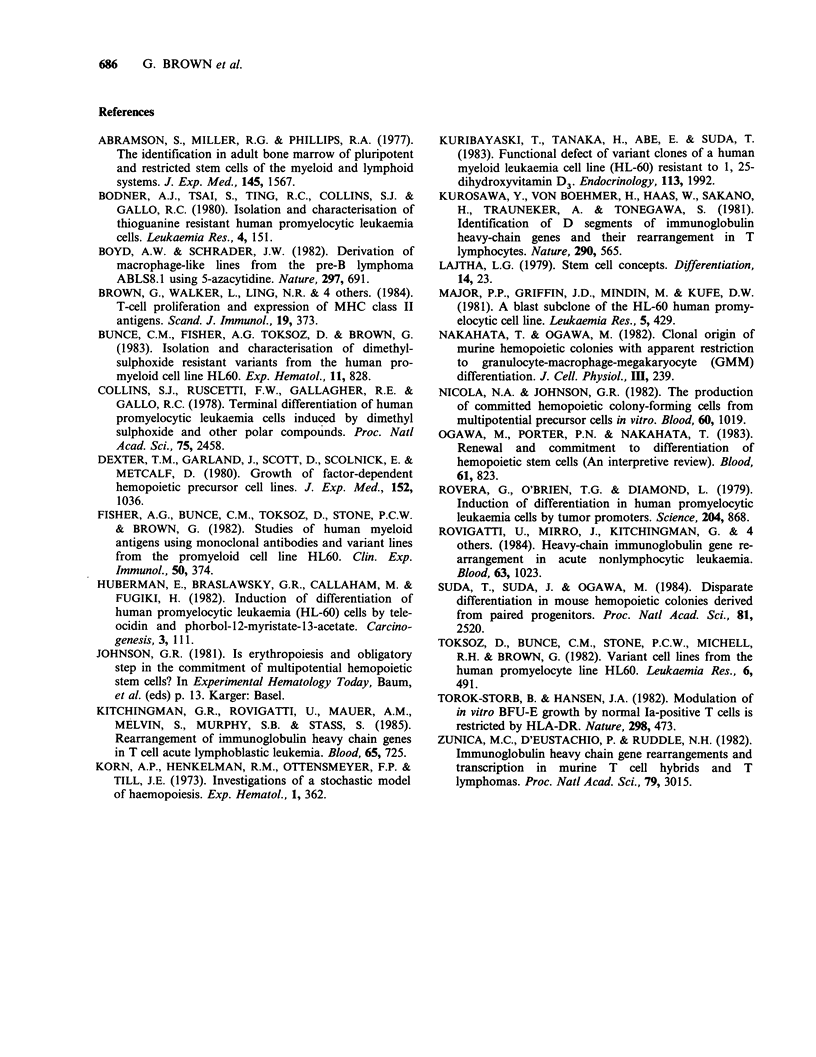# Sequential determination of lineage potentials during haemopoiesis.

**DOI:** 10.1038/bjc.1985.243

**Published:** 1985-11

**Authors:** G. Brown, C. M. Bunce, G. R. Guy


*Br. J. Cancer* (1985), **52**, 681–686

### Hypothesis

### Sequential determination of lineage potentials during haemopoiesis

G. Brown[1], C.M. Bunce[1] & G.R. Guy[2]

*Departments of [1]Immunology and [2]Biochemistry, University of Birmingham, Edgbaston, Birmingham B15 2TJ, UK.*

The haemopoietic system, in which proliferation and commitment to differentiation along at least five distinct pathways continues throughout life, provides an ideal cell system for studying mechanisms which direct cells to differentiate into one type or another. An insight into this problem is clearly important if we are to understand the population structure, kinetics and regulation of complex, heterogeneous cell populations such as the haemopoietic system. An understanding of these problems will be essential to resolve the problem of haemopoietic cell transformation which may often involve progenitor cells (Lajtha, 1979).

As yet, it is not clear how pluripotent haemopoietic stem cells give rise to monopotent progenitor cells. Ogawa and colleagues argue from their studies of oligopotent progenitor cells in culture that the restriction in differentiation potentials of stem cells is a progressive and stochastic process (Nakahata & Ogawa, 1982; Ogawa *et al.*, 1983; Suda *et al.*, 1984). Alternatively, Nicola and Johnson (1982) interpret their studies of committed progenitor cells to suggest 'a defined sequence of successive restriction of potentials'. In this case the pluripotent stem cells progressively loses differentiation options until only the capacity to differentiate along the erythroid lineage remains. We would like to consider a further model for stem cell commitment which hypothesizes that lineage potentials are consecutively and individually expressed in a defined sequence as progenitor cells mature. This possibility can be revealed from studies of variant lines derived from the promyeloid cell line HL60 described below.

HL60 cells are at a stage of maturation at which they are still able to differentiate into either neutrophils (Collins *et al.*, 1978) or monocytes (Rovera *et al.*, 1979) when treated with appropriate inducers. The bipotential nature of these cells provides a model system in which processes which restrict the differentiation of cells to a particular lineage can be studied. One useful approach to this problem is to derive variant sub-lines from HL60 cultures which show restricted abilities to mature towards either neutrophils and/or monocytes. We, together with other investigators, have described a number of variant HL60 lines (Bodner *et al.*, 1980; Major *et al.*, 1981; Fisher *et al.*, 1982; Huberman *et al.*, 1982; Toksoz *et al.*, 1982; Bunce *et al.*, 1983; Kuribayaski *et al.*, 1983). In our own studies stable sub-lines were isolated in medium containing 1.25% dimethylsulphoxide (DMSO) which optimally induces HL60 cells to differentiate into neutrophils. Cells of some, but not all of the sub-lines, fail to differentiate into monocytes when treated with either 12-0-tetradecanoylphorbol-13-acetate (TPA) or a factor used to induce monocyte differentiation (Bunce *et al.*, 1983; & unpublished observations). These spontaneously arising variant lines may represent subpopulations of cells within the parental HL60 culture with various inherent capacities for neutrophil and monocyte differentiation. The utility of these sub-lines lies in their contribution to our understanding of precisely how the variant cell populations relate to the parental cells and to each other. Do these lines represent cells at different stages of differentiation with respect to their ability to differentiate and their phenotypic characteristics?

An interesting correlation has been observed between the expression of two myeloid-associated antigens (AGF 4.36, AGF 4.48) and the ability of variant HL60 lines to differentiate towards monocytes. The variant lines which are unable to differentiate into monocytes express both myeloid antigens to the same extent as HL60. Conversely, lines able to mature towards monocytes fail to express these antigens (Bunce *et al.*, 1983). Both AGF 4.36 and AGF 4.48 antigens are lost as HL60 cells are induced to mature towards monocytes but only AGF 4.36 is lost during neutrophil differentiation (Fisher *et al.*, 1982; & see Figure 1). How can we now interpret both the inducibility of the variant cell lines and their expression of these myeloid antigens to give a cohesive picture of HL60 differentiation?

Collective data from our own studies of variant

Correspondence: G. Brown
Received 29 June 1985.

© The Macmillan Press Ltd., 1985

**Figure 1** A model for HL60 cell differentiation. Variant cell lines derived from HL60 are arranged in a proposed developmental sequence as regards their responsiveness to inducer of neutrophil and monocyte differentiation and the pattern of myeloid antigen expression by these lines.

HL60 lines (Toksoz *et al.*, 1982; Bunce *et al.*, 1983) and antigen expression during neutrophil and monocyte differentiation (Fisher *et al.*, 1982) are used to propose the model for HL60 differentiation shown in Figure 1. We assume in this figure that the variant sub-lines reflect an inherent heterogeneous responsiveness of HL60 cells to inducers of neutrophil and monocyte differentiation. The variant lines are arranged in a sequence of development which proposes that cells within HL60 cultures first acquire a capacity for differentiation induction into neutrophils; later they are able to differentiate into either neutrophils or monocytes and finally can only be directed towards monocyte differentiation. As observed experimentally, this inherent developmental progression does not continue spontaneously to give rise to large numbers of terminally differentiated cells. The culture habitat in some way attains a steady-state balance covering a range of potential for both neutrophil and monocyte differentiation. The variant lines HL60 Ast 3, Ast 4 and Spl mostly represent cells within the parental HL60 culture which have not yet undergone a differentiation step necessary for acquisition of the ability to respond to induction signals for monocyte differentiation. These lines are unresponsive to a wide range of concentrations of inducers of monocyte differentiation. The HL60 Ast 3, Ast 4 and Spl lines fail to respond to TPA concentrations in the range 4–50 nM as compared with 4 nm TPA required to induce HL60 monocyte differentiation (Bunce *et al.*, 1983). Similarly, conditioned medium containing a factor which induces monocyte differentiation is used at a concentration of 2.5% to induce HL60 differentiation and the HL60 Ast 3, Ast 4 and Spl lines respond minimally to concentrations of medium of up to 10% (unpublished observations). The variant lines HL60m2, m4, Ast 25 and Ast 1 have properties which indicate they are past the differentiation step at which cells are optimally bipotential. Figure 1 shows there is first a progressive acquisition of capacity to be induced to neutrophil differentiation within cells in HL60 cultures followed by a progressive loss of this capacity as cells are subsequently restricted to monocyte differentiation. It is proposed that the variant lines which require higher concentrations of DMSO than the parental line to be induced to differentiate towards neutrophils reflect earlier and later phases of maturation. Neutrophil differentiation is induced in HL60 cultures by 1.25% DMSO. HL60m2, m4, Ast 3 and Spl can be induced to mature into neutrophils using 1.75% DMSO, HL60 Ast 25 cells require 2.0% DMSO and HL60 Ast 1 and Ast 4 cultures show minimal neutrophil differentiation at a concentration of 2.0% DMSO.

The picture which emerges is that the HL60 and variant cells populations as a whole maintain different steady-states as regards the distribution of cells at various stages of commitment to neutrophil and/or monocyte differentiation. An indication that a shift in the distribution of committed cells and hence differentiation status has occurred in the variant cell cultures is shown by the following observation. The HL60m2 and m4 lines are shown in Figure 1 to be more mature than HL60 cells with respect to their progress towards monocyte differentiation. These cell lines show to a minor degree some spontaneous differentiation into monocytes even when cultured routinely in 1.25% DMSO. Occasional alpha-napthylbutyrate esterase positive cells are routinely seen in HL60m2 and m4 cultures. These are not observed in HL60 cultures.

If the variant cell lines are arranged in the proposed developmental sequence the pattern of myeloid antigen expression by these lines is found to correlate with antigen expression when HL60 is induced to differentiate to neutrophils or monocytes. Both myeloid antigens, AGF 4.36 and AGF 4.48, which are lost during monocyte differentiation are not expressed by the variant cell lines which to some extent are more inclined towards monocyte differentiation. Variant lines showing a reduced ability for neutrophil differentiation, but at a stage prior to acquiring the potential for monocyte differentiation, express both antigens one of which is retained throughout neutrophil differentiation. It is interesting to look at expression of the AGF 4.48 antigen, which is normally retained throughout HL60 neutrophil differentiation, as the HL60m2 and m4 cells mature towards neutrophils when treated with 1.75% DMSO. If these lines are correctly placed in the developmental sequence and are at a stage of having lost the AGF 4.48 antigen (rather than at a stage prior to expressing this antigen) and the developmental program is irreversible then HL60m2 and m4 cells should mature into neutrophils without expressing the AGF 4.48 antigen. Neither the AGF 4.48 nor the AGF 4.36 antigen is expressed as HL60m2 and m4 cells mature into neutrophils (Bunce *et al.*, 1983).

Cells within the HL60 population show the same sequence of acquisition of neutrophil and monocyte differentiation capacity as proposed during normal myelopoiesis. Studies of Dexter and colleagues have shown that normal progenitor cells first acquire the capacity to be induced to terminally differentiate to neutrophils and subsequently acquire responsiveness to inducers of monocyte differentiation. Thus, the proposed sequence of development of committed progenitor cells during normal neutrophil and macrophage differentiation is granulocyte-colony forming cell(CFC)→granulocyte/macrophage-CFC→ macrophage-CFC (Dexter *et al.*, 1980). It is interesting to speculate whether a progressive acquisition and restriction to neutrophil development followed by loss of this potential as progenitor cells are restricted to monocyte differentiation occurs only in relation to granulocyte/macrophage progenitor cells or reveals a fundamental principle during haemopoiesis. This sequential process of stem cell commitment is contrary to the 'stochastic' model of Ogawa *et al.* (1983) and 'successive restriction' model of Nicola and Johnson (Nicola & Johnson, 1982).

In Figure 2 we propose that as pluripotent stem cells are committed to differentiation their future possible goals are individually and sequentially expressed in an order determined inherently within the genome. Cells within the sequence shown are precommitted as regards their ability to respond to various inducers of differentiation and on encountering an appropriate factor(s) or suitable microenvironment undergo proliferation and maturation along a particular pathway. Progenitor cells which do not receive a signal for differentiation towards mature end cells progress onto the next stages in the sequence of development. Commitment is gradual so that the progenitor cells are a continuous spectrum of cells. Therefore cells may be at a developmental stage of being able to respond, for example, to either inducers of neutrophil or monocyte differentiation, as is the case of HL60 cells. In terms of renewal of progenitor cell populations, cells continuously occupy each potential for differentiation at any given time and therefore respond to the requirement for each mature cell type. The continuous development of progenitor cells may not be diverted entirely towards, for example, granulocyte production in the presence of granulocyte differentiation factors for the reasons that progenitor cells which respond, as they divide, may generate some cells which are still able to progress or are selected to progress to the next stage of commitment. This decision may be governed by a stochastic rule in view of the data of Ogawa and colleagues (Ogawa *et al.*, 1983; Suda *et al.*, 1984) and Korn and colleagues (Korn *et al.*, 1973).

There is no direct evidence for the order of cell commitment shown in Figure 2. However, a number of diverse observations support the proposed sequence. The transition from granulocyte commitment to monocyte commitment has been argued from studies of variant cell lines derived from HL60. A close relationship between the potentials for macrophage and B cell differentiation can be inferred from the experiments of Boyd and Schrader (1982). In their studies cloned macrophage-like cell lines were derived from the murine pre-B lymphoma ABLS8.1 after cells were exposed to 5-azacytidine. As in the studies of HL60 described above, it is possible that the potential for monocyte differentiation revealed by the sub-lines represents heterogeneity within the parental cell population. In the case of both the HL60 and

**Figure 2** Proposed sequence of cell commitment during haemopoiesis. The cells diagonally to the left represent progenitor cells committed to megakaryocyte (Me), erythroid (E), neutrophil (G), monocyte (M), B cell (B) and T cell (T) differentiation. Differentiation towards mature end cells is indicated by → and the process of progenitor cell development by ⇝.

ABLS8.1 cell lines, the arguments assume that the differentiation potentials observed within ABLS8.1 or when HL60 cells are induced to mature are representative of the developmental relationships which occur in normal progenitor cells. Problems associated with aneuploidy and gene amplification effects could argue that cultured tumour cell lines may give misleading information. Nevertheless, it is likely that the normal sequence of development of lineage potentials is maintained in many tumour cell lines.

Studies of rearrangement of immunoglobulin heavy chain genes in the human myeloid cell line ML-1 (Rovigatti *et al.*, 1984), murine T cell hybrids and T lymphomas (Zunica *et al.*, 1982) and T cell acute lymphoblastic leukaemia (Kitchingman *et al.*, 1985) support a sequence of progression from myeloid to B cell and subsequent T-cell commitment. In the case of ML-1, rearrangements in the heavy chain region suggest that this cell line represents transformation of a progenitor cell with potentials for both myeloid and B cell differentiation (Rovigatti *et al.*, 1984). Kurosawa and colleagues suggested from their early findings that diversity (D) and joining ($J_H$) segments of immunoglobulin heavy chain genes are joined in DNA from cloned murine cytolytic T cells that this may have occurred in a progenitor cell which is common to both B and T cells (Kurosawa *et al.*, 1981). Whether commitment to B and T cell differentiation represents the last stages in the developmental sequence is at present unclear. An alternative viewpoint is that potentials for B and T cell differentiation are expressed prior to the sequence of megakaryocyte, erythroid and granulocyte commitment. This is suggested by the studies of Abramson and colleagues of radiation-induced chromosome aberrations in progenitor cells which proposed that B lymphocytes are derived directly from the pluripotent stem cell (Abramson *et al.*, 1977).

The proposed sequence of commitment at early stages from megakaryocyte to erythroid to neutrophil potentials takes into considerations the lineage potentials of progenitor cells in culture (CFC) which appear to be restricted to two pathways of differentiation. Megakaryocyte/erythroid and erythroid/neutrophil progenitor cells have been described in cultures of human and murine cells (Ogawa *et al.*, 1983). However, in this case and also situations where maturation along four lineages is observed i.e. granulocyte-erthrocyte-macrophage-megakaryocyte (GEMM) colonies, the types of cells found in colonies in culture may depend on the relative presence of factors which support expression of various potentialities or progression along the sequence of commitment. As in the model of Nicola and Johnson (1982) commitment to megakaryocyte and erythroid differentiation are obligatory steps during stem cell maturation and erythroid cells together with megakaryocytes should always be seen in mixed colonies. Erythroid cells are invariably seen (Johnson, 1981) and colonies would have to be stained appropriately to show the presence of megakaryocytes (Nicola & Johnson, 1982).

The linear progression of events during stem cell commitment can be considered either in terms of the sequential ability of cells to respond to various inducers of differentiation or that there is a predetermined sequential rather than random rearrangement of various genes prerequisite for differentiation towards a particular cell type. In this case, for example, during B and T cell development, the progenitor cell first rearranges immunoglobulin heavy chain genes which is prerequisite though insufficient for terminal B cell differentiation (Kitchingman *et al.*, 1985). Failure to receive a signal required to continue development along this pathway leads then to rearrangement of receptor genes appropriate for T cell maturation.

The model proposed has important implications as regards malignancy and the control of normal haemopoiesis. At present, it is not known whether gene rearrangement occurs in cells other than lymphocytes which clearly rearrange genes with respect to receptor diversity. However, progenitor cells together with lymphocytes are often 'targets' for cell transformation which can be explained by suggesting that DNA rearrangement during stem cell determination and lymphocyte differentiation provides active sites within the genome which are susceptible to inappropriate gene recombination events. Hence, during normal cell commitment patterns of gene rearrangement are conservatively followed. Some genetic processes may be repeated if they are useful at each stage of development, for example, genomic events which determine at each state of commitment to what extent cells are able to proliferate. Leukaemias, other than those involving mature, immunocompetent T or B cells, may be viewed as a lesion in the progressive events during stem cell determination. If inappropriate gene rearrangement occurs late in the sequence of development of progenitor cells then this gives rise to the acute lymphoid leukaemias. Chronic myeloid leukaemia could be viewed as the same lesion occurring at a very early stage in progenitor cell development which is manifest throughout sequential commitment of the 'transformed' clone.

The maturation sequence of progenitor cells is pertinent to considerations of the nature of surface molecules and factors which regulate the proliferation and development of committed cells. Cell surface molecules which are expressed throughout the stages of commitment, for example, class II antigens, which are present on erythroid, myeloid and lymphoid precursor cells, may have a functional role in controlling the proliferation or differentiation status of cells during the process of cell commitment (Brown *et al.*, 1984). At the end of the sequence shown in Figure 2 progenitor cells committed to T cell differentiation are the last to be generated. Torok-Storb and Hansen (1982) have shown that T cells can enhance and limit the growth of erythroid progenitor cells. Class II molecules seem to be involved in the inhibiting effect of T cells. It is interesting to speculate that T cells may provide an important feedback control on the rate of renewal of progenitor cells. Also, it is important to consider whether interleukins such as IL3 are required during sequential determination of lineage potentials if this process involves cell divisions.

To return to the problem of how cells mature into one cell type or another, it is likely that the changes occurring are gradual and progressive, as opposed to clear on/off, events within cells. This makes analysis a little more difficult but resolution is possible by using populations of cells at various stages in a particular developmental sequence. The variant HL60 lines, in the order show in Figure 1, essentially typify the progression of acquisition of neutrophil and monocyte differentiation capacity. Analysis of events which direct HL60 cells to differentiate into one cell type or another can be approached by looking for intrinsic patterns of differences between the variant lines which correlate with their relative position in the developmental sequence.

We thank the Leukaemia Research Fund for support of research in our laboratory and Paula Brown for typing the manuscript.

### References

ABRAMSON, S., MILLER, R.G. & PHILLIPS, R.A. (1977). The identification in adult bone marrow of pluripotent and restricted stem cells of the myeloid and lymphoid systems. *J. Exp. Med.*, **145**, 1567.

BODNER, A.J., TSAI, S., TING, R.C., COLLINS, S.J. & GALLO, R.C. (1980). Isolation and characterisation of thioguanine resistant human promyelocytic leukaemia cells. *Leukaemia Res.*, **4**, 151.

BOYD, A.W. & SCHRADER, J.W. (1982). Derivation of macrophage-like lines from the pre-B lymphoma ABLS8.1 using 5-azacytidine. *Nature*, **297**, 691.

BROWN, G., WALKER, L., LING, N.R. & 4 others. (1984). T-cell proliferation and expression of MHC class II antigens. *Scand. J. Immunol.*, **19**, 373.

BUNCE, C.M., FISHER, A.G. TOKSOZ, D. & BROWN, G. (1983). Isolation and characterisation of dimethylsulphoxide resistant variants from the human promyeloid cell line HL60. *Exp. Hematol.*, **11**, 828.

COLLINS, S.J., RUSCETTI, F.W., GALLAGHER, R.E. & GALLO, R.C. (1978). Terminal differentiation of human promyelocytic leukaemia cells induced by dimethyl sulphoxide and other polar compounds. *Proc. Natl Acad. Sci.*, **75**, 2458.

DEXTER, T.M., GARLAND, J., SCOTT, D., SCOLNICK, E. & METCALF, D. (1980). Growth of factor-dependent hemopoietic precursor cell lines. *J. Exp. Med.*, **152**, 1036.

FISHER, A.G., BUNCE, C.M., TOKSOZ, D., STONE, P.C.W. & BROWN, G. (1982). Studies of human myeloid antigens using monoclonal antibodies and variant lines from the promyeloid cell line HL60. *Clin. Exp. Immunol.*, **50**, 374.

HUBERMAN, E., BRASLAWSKY, G.R., CALLAHAM, M. & FUGIKI, H. (1982). Induction of differentiation of human promyelocytic leukaemia (HL-60) cells by teleocidin and phorbol-12-myristate-13-acetate. *Carcinogenesis*, **3**, 111.

JOHNSON, G.R. (1981). Is erythropoiesis and obligatory step in the commitment of multipotential hemopoietic stem cells? In *Experimental Hematology Today*, Baum, *et al.* (eds) p. 13. Karger: Basel.

KITCHINGMAN, G.R., ROVIGATTI, U., MAUER, A.M., MELVIN, S., MURPHY, S.B. & STASS, S. (1985). Rearrangement of immunoglobulin heavy chain genes in T cell acute lymphoblastic leukemia. *Blood*, **65**, 725.

KORN, A.P., HENKELMAN, R.M., OTTENSMEYER, F.P. & TILL, J.E. (1973). Investigations of a stochastic model of haemopoiesis. *Exp. Hematol.*, **1**, 362.

KURIBAYASKI, T., TANAKA, H., ABE, E. & SUDA, T. (1983). Functional defect of variant clones of a human myeloid leukaemia cell line (HL-60) resistant to 1, 25-dihydroxyvitamin $D_3$. *Endocrinology*, **113**, 1992.

KUROSAWA, Y., VON BOEHMER, H., HAAS, W., SAKANO, H., TRAUNEKER, A. & TONEGAWA, S. (1981). Identification of D segments of immunoglobulin heavy-chain genes and their rearrangement in T lymphocytes. *Nature*, **290**, 565.

LAJTHA, L.G. (1979). Stem cell concepts. *Differentiation*, **14**, 23.

MAJOR, P.P., GRIFFIN, J.D., MINDIN, M. & KUFE, D.W. (1981). A blast subclone of the HL-60 human promyelocytic cell line. *Leukaemia Res.*, **5**, 429.

NAKAHATA, T. & OGAWA, M. (1982). Clonal origin of murine hemopoietic colonies with apparent restriction to granulocyte-macrophage-megakaryocyte (GMM) differentiation. *J. Cell. Physiol.*, **III**, 239.

NICOLA, N.A. & JOHNSON, G.R. (1982). The production of committed hemopoietic colony-forming cells from multipotential precursor cells *in vitro*. *Blood*, **60**, 1019.

OGAWA, M., PORTER, P.N. & NAKAHATA, T. (1983). Renewal and commitment to differentiation of hemopoietic stem cells (An interpretive review). *Blood*, **61**, 823.

ROVERA, G., O'BRIEN, T.G. & DIAMOND, L. (1979). Induction of differentiation in human promyelocytic leukaemia cells by tumor promoters. *Science*, **204**, 868.

ROVIGATTI, U., MIRRO, J., KITCHINGMAN, G. & 4 others. (1984). Heavy-chain immunoglobulin gene rearrangement in acute nonlymphocytic leukaemia. *Blood*, **63**, 1023.

SUDA, T., SUDA, J. & OGAWA, M. (1984). Disparate differentiation in mouse hemopoietic colonies derived from paired progenitors. *Proc. Natl Acad. Sci.*, **81**, 2520.

TOKSOZ, D., BUNCE, C.M., STONE, P.C.W., MICHELL, R.H. & BROWN, G. (1982). Variant cell lines from the human promyelocyte line HL60. *Leukaemia Res.*, **6**, 491.

TOROK-STORB, B. & HANSEN, J.A. (1982). Modulation of *in vitro* BFU-E growth by normal Ia-positive T cells is restricted by HLA-DR. *Nature*, **298**, 473.

ZUNICA, M.C., D'EUSTACHIO, P. & RUDDLE, N.H. (1982). Immunoglobulin heavy chain gene rearrangements and transcription in murine T cell hybrids and T lymphomas. *Proc. Natl Acad. Sci.*, **79**, 3015.